# Supplementary material for: GSA: an independent development algorithm for calling copy number and detecting homologous recombination deficiency (HRD) from target capture sequencing
Source: BMC Bioinformatics. 2021 Nov 23;22:562. doi: 10.1186/s12859-021-04487-9 (PMC8609767; doi:10.1186/s12859-021-04487-9)
Supplement: Supplementary file 1 — Additional file 1. Supplementary material. Additional figures and tables to support on the understanding the GSA algorithm. [file 12859_2021_4487_MOESM1_ESM.pdf]

Supplementary Material Captions

Supplementary Fig. 1 The schematic diagram for tree recursion (TR) segmentation algorithm.

Supplementary Fig. 2 The bioinformation analysis flow chart of the GSA algorithm.

Supplementary Fig. 3 The ROC curve modelled by 62 *BRCA1/2*-deficiency samples and 37 *BRCA1/2* wildtype clinical samples of the 195 patients.

Supplementary Fig. 4 The comparison diagram between before and after removing the abnormal segments according to BAF and LRR. (A) Before removing the abnormal segments according to BAF. (B) After removing the abnormal segments according to BAF. (C) Before removing the abnormal segments according to LRR. (D) After removing the abnormal segments according to LRR.

Supplementary Fig. 5 The consistency of HRD score calculated by the GSA algorithm with different purity for 3 cell lines (A) and 4 clinical samples (B).

Supplementary Fig. 6 The BAF and CN mapping of HCC38 with high HRD score.

Supplementary Fig. 7 The BAF and CN mapping of ZR-75-30 with low HRD score.

Supplementary Fig. 8 The waterfall map of 15 HRR pathway genes that mutated in the 195 clinical samples. The annotation of mutation types was shown on the upper right with various colors. The samples were sorted by HRD scores.

Supplementary Fig. 9 The chromosome segmentation effects comparison of GSA, PureCN and ASCAT algorithms in chromosome 2.

Supplementary Table 1 The relationship between theoretical copy number with tumor purity at specific genotype.

Supplementary Table 2 The relationship between theoretical BAF with tumor purity at specific genotype.

Supplementary Table 3 The HRD score and BRCA1/2 deficiency status of 195 clinical samples.

Supplementary Table 4 Tumor purity and ploidy values calculated by clinical samples and tumor cell lines with different tumor purity.

Supplementary Table 5 Comparison of the chromosome segmentation and copy number of GSA, PureCN, and ASCAT.

Supplementary Table 6 Comparison of the tumor purity and ploidy predicted by GSA, PureCN, and ASCAT.

Supplementary Fig. 1 The schematic diagram for tree recursion (TR) segmentation algorithm.

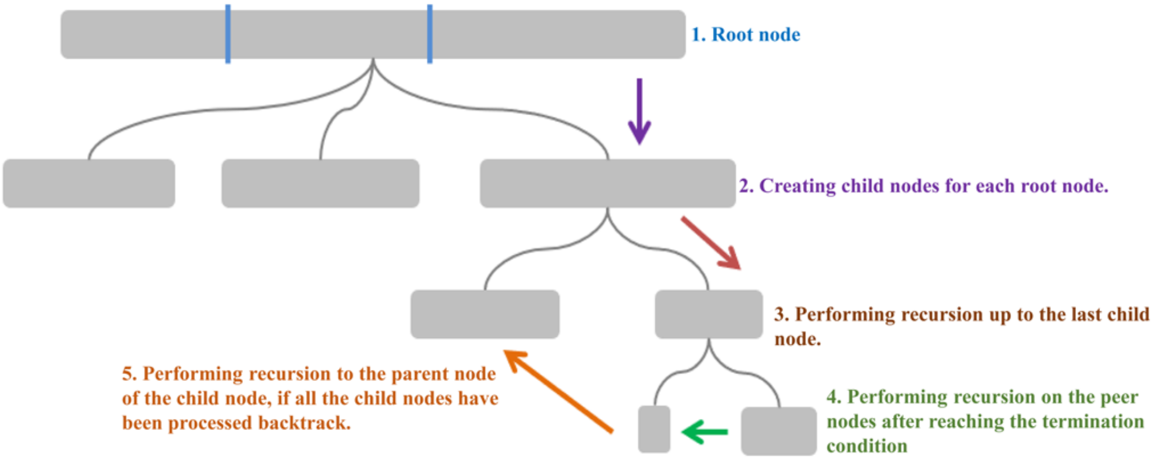

Supplementary Fig. 2 The bioinformation analysis flow chart of the GSA algorithm.

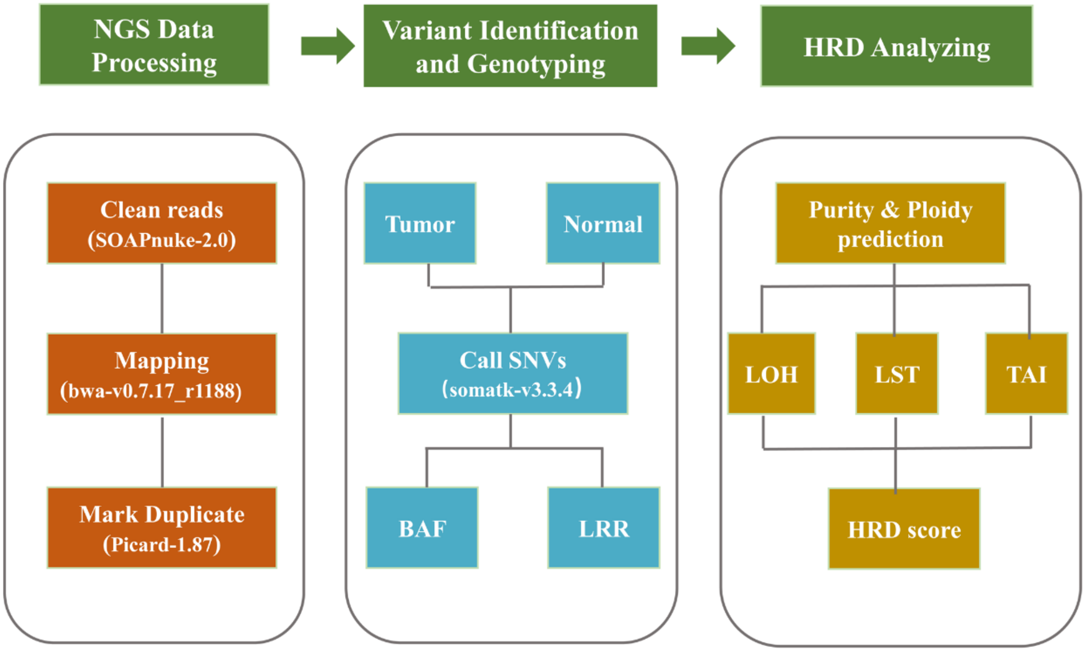

Supplementary Fig. 9 The ROC curve modelled by 62 *BRCA1/2*-deficiency samples and 37 *BRCA1/2* wildtype clinical samples of the 195 patients.

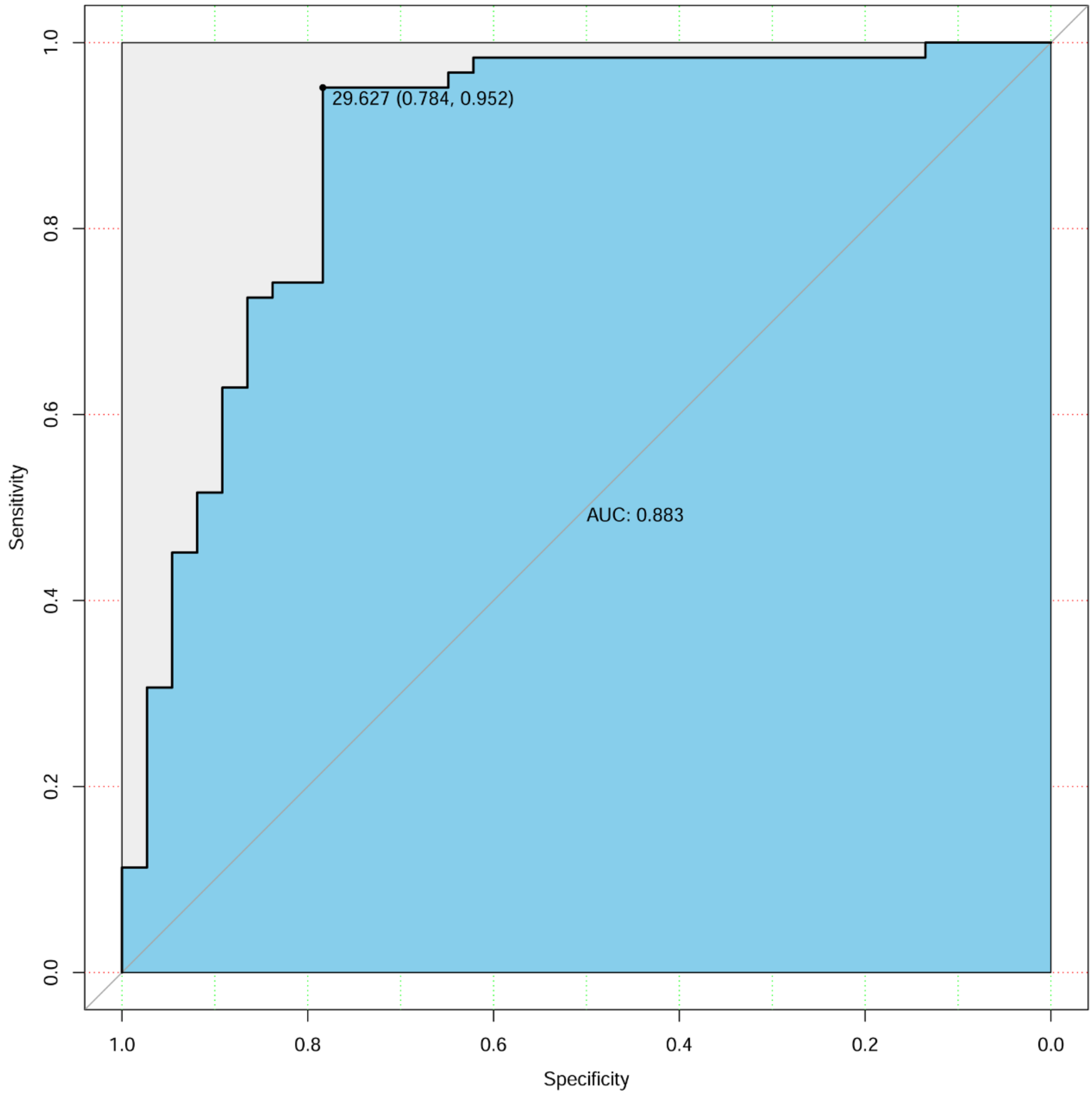

Supplementary Fig. 4 The comparison diagram between before and after removing the abnormal segments according to BAF and LRR. (A) Before removing the abnormal segments according to BAF. (B) After removing the abnormal segments according to BAF. (C) Before removing the abnormal segments according to LRR. (D) After removing the abnormal segments according to LRR.

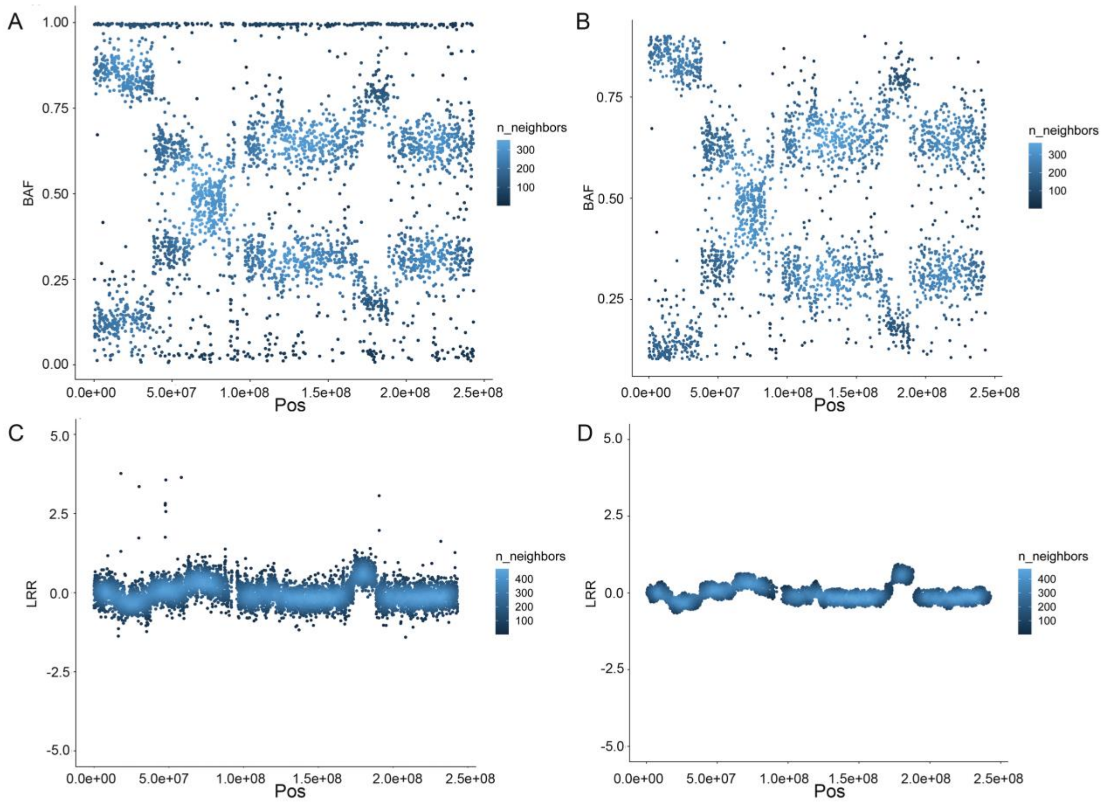

Supplementary Fig. 5 The consistency of HRD score calculated by the GSA algorithm with different purity for 3 cell lines (A) and 4 clinical samples (B).

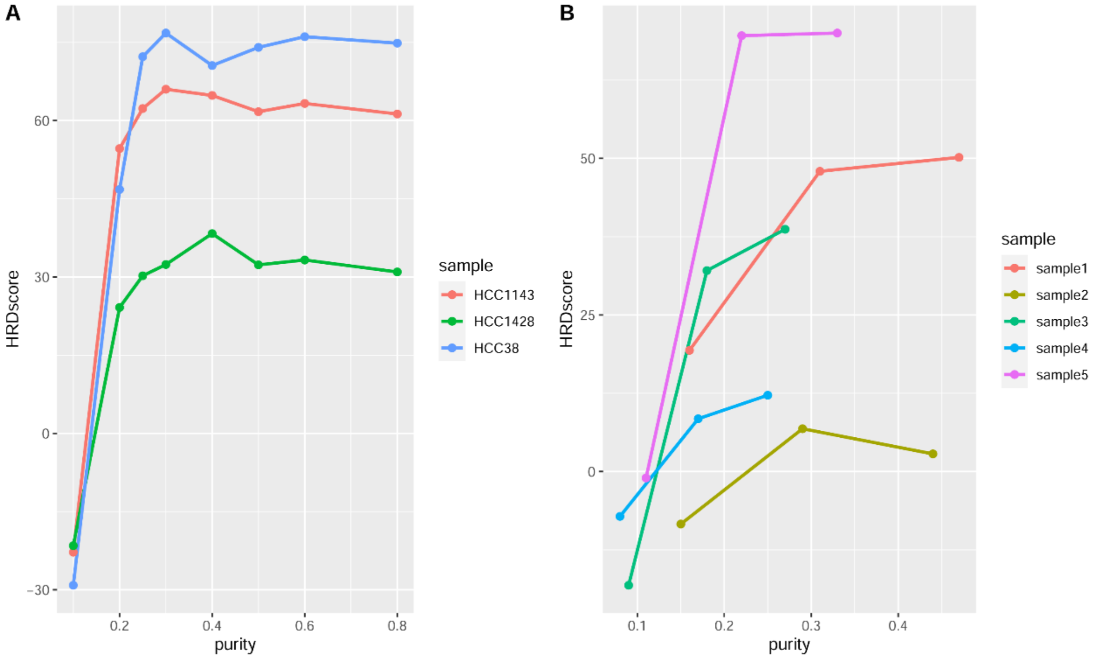

Supplementary Fig. 6 The BAF and CN mapping of HCC38 with high HRD score.

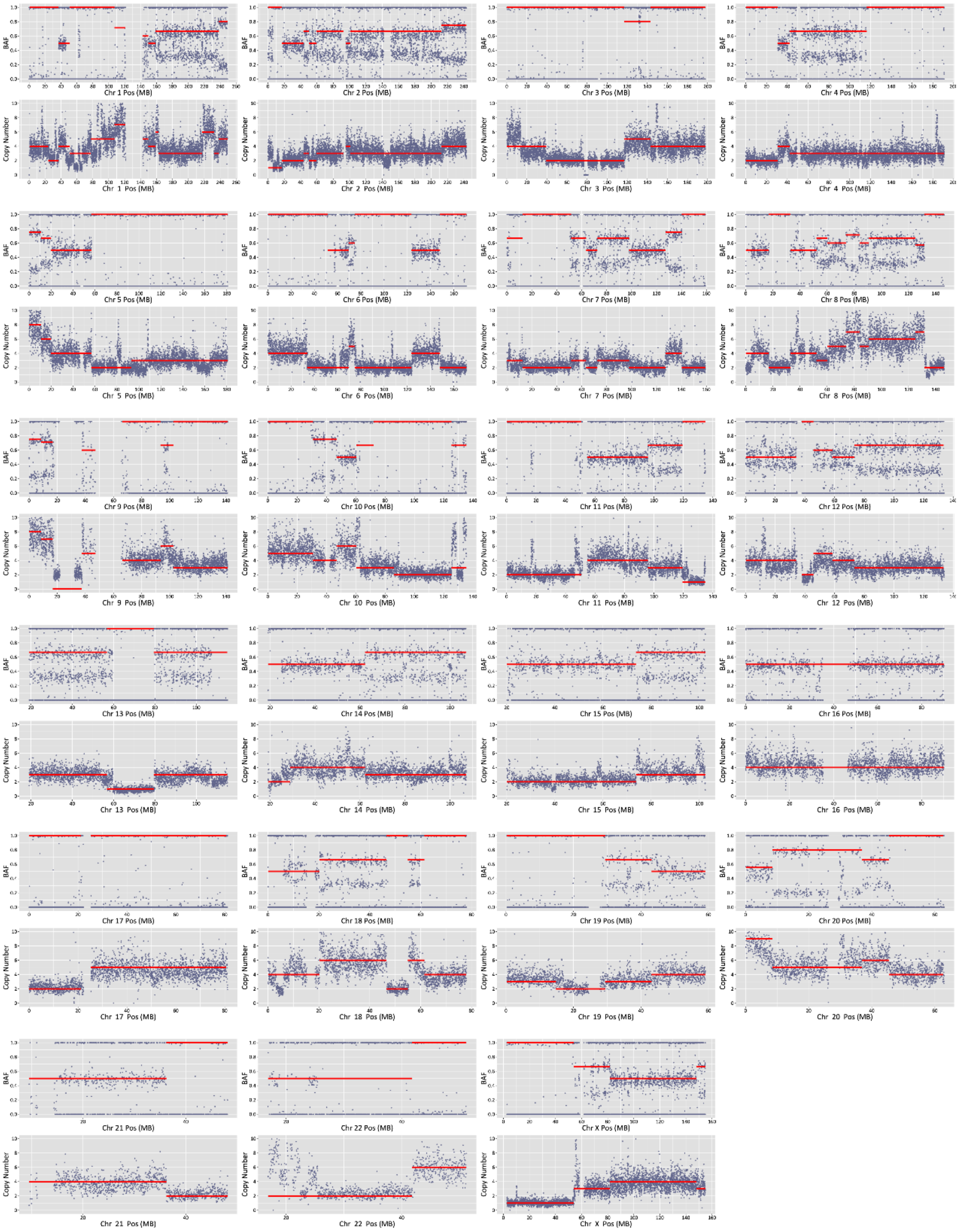

Supplementary Fig.7 The BAF and CN mapping of ZR-75-30 with low HRD score.

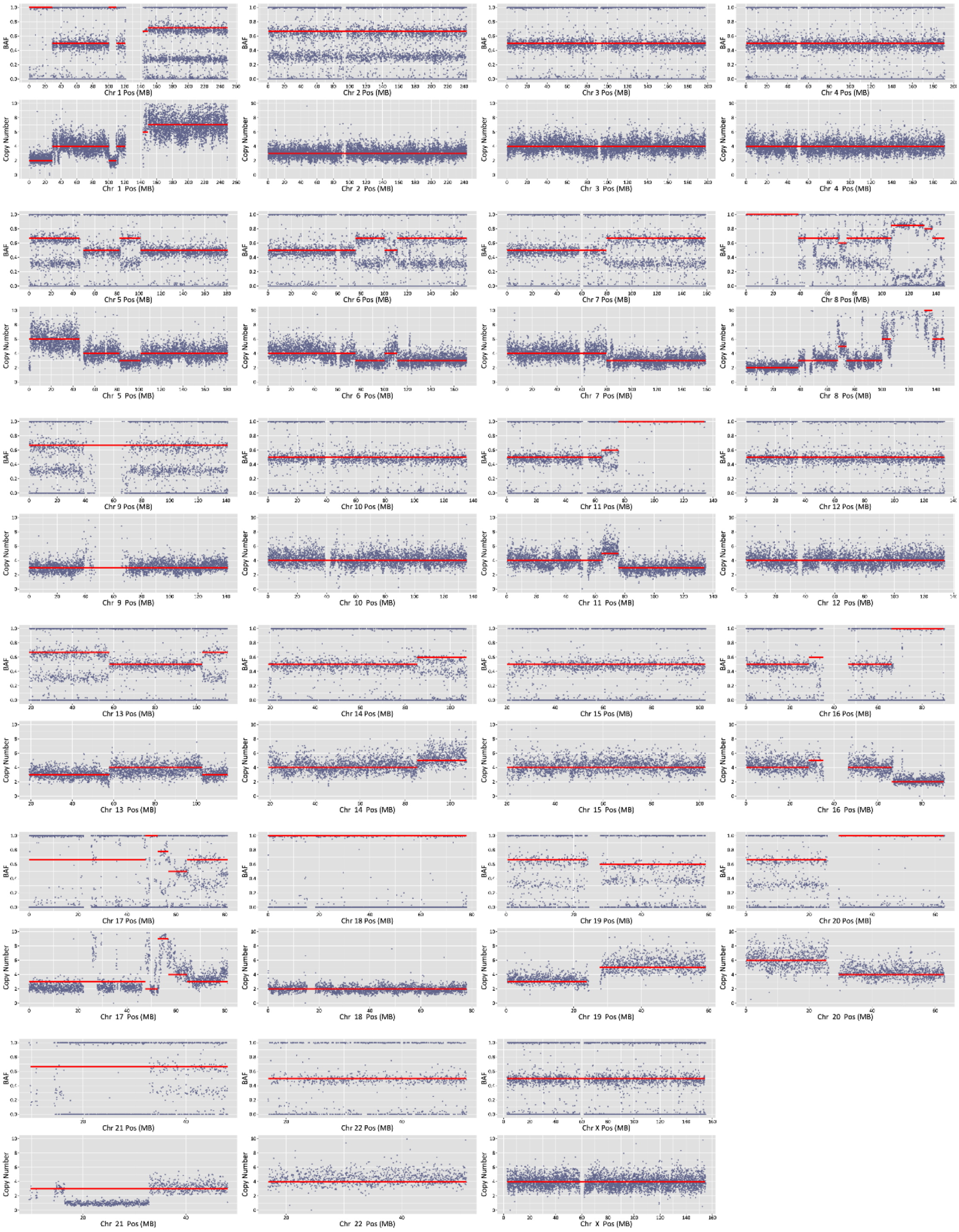

Supplementary Fig. 8 The waterfall map of 15 HRR pathway genes that mutated in the 195 clinical samples. The annotation of mutation types was shown on the upper right with various colors. The samples were sorted by HRD scores.

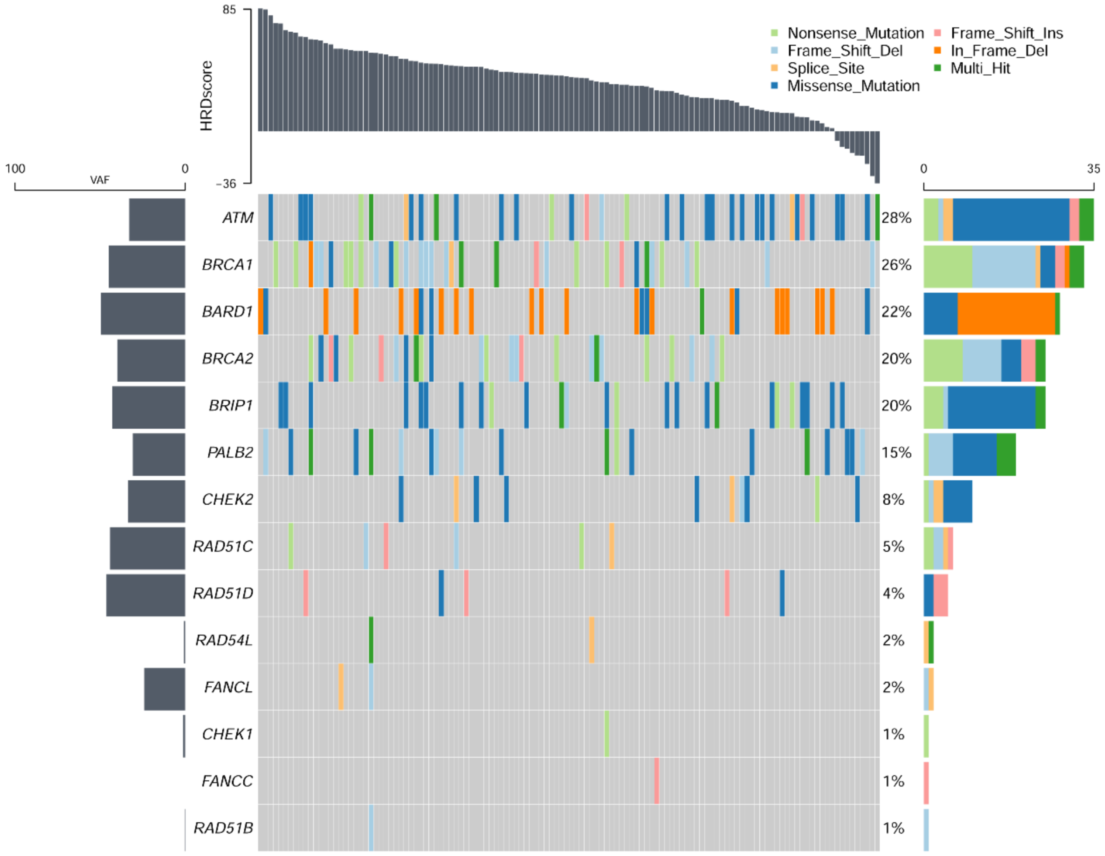

Supplementary Fig. 9 The chromosome segmentation effects comparison of GSA, PureCN and ASCAT algorithms in chromosome 2.

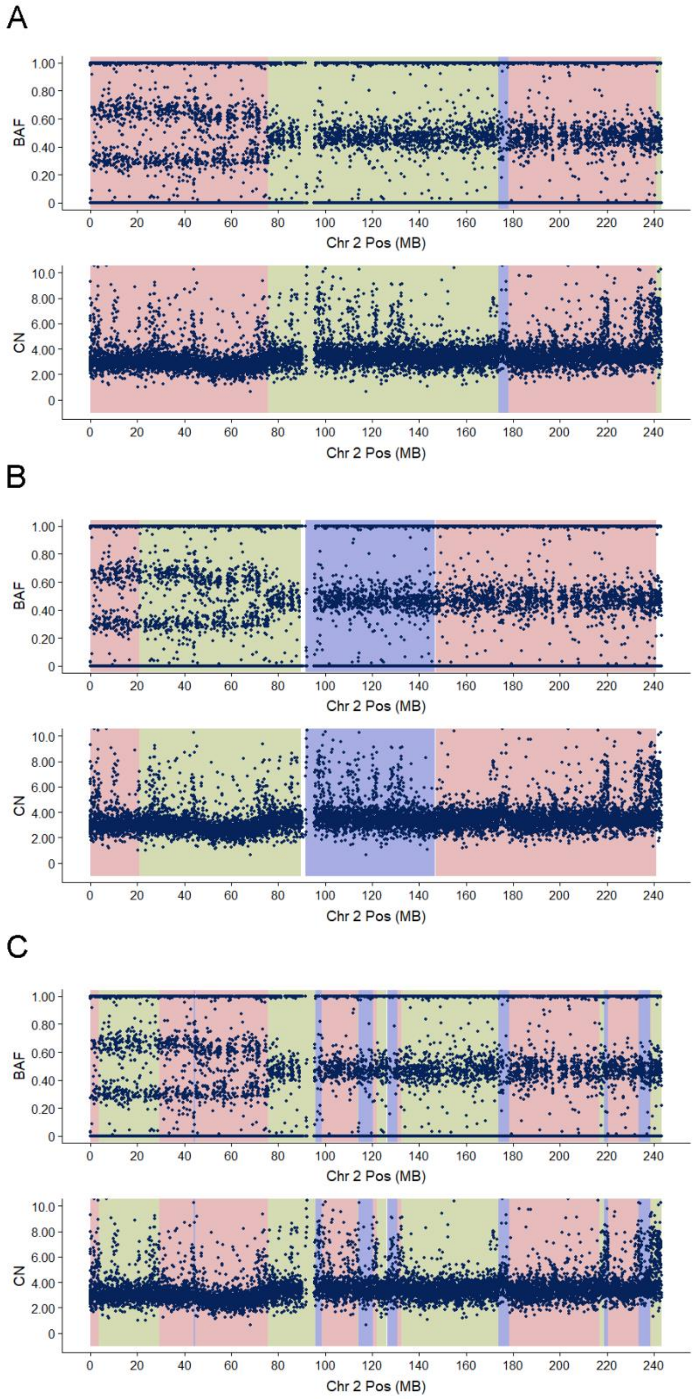

Supplementary Table 1 The relationship between theoretical copy number with different tumor purity at specific genotype.

Tumor purity	Theoretical copy number of a specific genotype										
	B	AB	BB	ABB	BBB	AABB	ABBB	BBBB	AABBB	ABBBB	BBBBB
100%	1.0	2.0	2.0	2.0	3.0	4.0	4.0	4.0	5.0	5.0	5.0
80%	1.2	2.0	2.0	2.8	2.8	3.6	3.6	3.6	4.4	4.4	4.4
60%	1.4	2.0	2.0	2.6	2.6	3.2	3.2	3.2	3.8	3.8	3.8
40%	1.6	2.0	2.0	2.4	2.4	2.8	2.8	2.8	3.2	3.2	3.2
20%	1.8	2.0	2.0	2.2	2.2	2.4	2.4	2.4	2.6	2.6	2.6

Supplementary Table 2 The relationship between theoretical BAF with different tumor purity at specific genotype.

Tumor purity	Theoretical BAF value of a specific genotype										
	B	AB	BB	ABB	BBB	AABB	ABBB	BBBB	AABBB	ABBBB	BBBBB
100%	1.00	0.50	1.00	0.67	1.00	0.50	0.75	1.00	0.60	0.80	1.00
80%	0.83	0.50	0.90	0.64	0.93	0.50	0.72	0.94	0.59	0.77	0.96
60%	0.71	0.50	0.80	0.62	0.85	0.50	0.69	0.88	0.58	0.74	0.90
40%	0.63	0.50	0.70	0.58	0.75	0.50	0.64	0.79	0.56	0.69	0.81
20%	0.56	0.50	0.60	0.55	0.64	0.50	0.58	0.67	0.54	0.62	0.69

Supplementary Table 3 The HRD score and *BRCA1/2* deficiency status of 195 clinical samples

Sample	Cancer Type	Purity	LOH	TAI	LST	Ploidy	HRD Score	BRCA Deficiency Status
S001	OC	0.44	34	18	80	3.31	80.70	N
S002	OC	0.41	29	18	63	2.66	68.77	Y
S003	OC	0.75	31	20	42	1.95	62.78	N
S004	OC	0.69	33	18	41	1.92	62.24	N
S005	OC	0.79	22	19	71	3.23	61.94	N
S006	OC	0.53	19	19	76	3.64	57.58	Y
S007	OC	0.41	28	21	36	1.94	54.93	N
S008	OC	0.70	28	21	32	1.78	53.41	N
S009	OC	0.51	9	19	78	3.70	48.65	N
S010	OC	0.31	24	19	34	1.99	46.16	N
S011	OC	0.62	24	12	39	1.91	45.40	N
S012	OC	0.64	21	16	36	1.94	42.93	N
S013	OC	0.45	22	22	48	3.28	41.16	Y
S014	OC	0.53	13	16	60	3.12	40.64	Y
S015	OC	0.72	23	14	32	1.87	40.02	Y
S016	OC	0.46	17	9	62	3.14	39.33	N
S017	OC	0.83	20	16	32	1.88	38.86	Y
S018	OC	0.55	18	20	55	3.59	37.36	Y
S019	OC	0.37	23	18	43	3.04	36.88	N
S020	OC	0.67	19	16	29	2.03	32.54	N
S021	OC	0.70	14	18	53	3.39	32.46	Y
S022	OC	0.67	17	14	55	3.71	28.50	N
S023	OC	0.26	17	15	37	2.61	28.55	N
S024	OC	0.66	17	17	26	1.92	30.24	Y
S025	OC	0.58	16	14	24	1.81	25.95	N
S026	OC	0.43	7	18	57	3.66	25.27	N
S027	OC	0.88	15	19	29	2.01	31.84	Y
S028	OC	0.83	16	11	26	1.94	22.93	N
S029	OC	0.21	17	9	37	2.65	21.93	N
S030	OC	0.83	17	12	23	1.94	21.93	Y
S031	OC	0.79	18	11	23	1.94	21.93	N
S032	OC	0.68	18	8	25	2.01	19.85	N
S033	OC	0.67	18	11	35	2.92	18.74	N
S034	OC	0.65	15	7	21	1.69	16.81	N
S035	OC	0.39	13	9	24	2.06	14.07	N
S036	OC	0.77	6	15	35	2.84	11.98	N
S037	OC	0.83	5	2	6	1.91	-16.61	N
S038	OC	0.43	4	2	10	2.12	-16.86	N
S039	OC	0.34	2	1	8	2.33	-25.12	N
S040	OC	1.00	0	0	0	2.00	-31.00	N
S041	OC	0.78	29	24	75	2.75	85.38	Y

S042	OC	0.89	41	24	46	1.69	84.81	Y
S043	OC	0.60	37	24	41	1.65	76.43	Y
S044	BC	0.41	36	64	15	2.57	75.17	N
S045	OC	0.56	34	19	64	2.71	75.00	Y
S046	OC	0.63	34	49	17	1.90	70.55	N
S047	OC	0.29	29	14	58	1.98	70.31	Y
S048	OC	0.52	32	23	42	1.79	69.26	Y
S049	OC	0.78	32	22	39	1.74	66.03	N
S050	OC	0.70	27	23	57	2.66	65.77	N
S051	BC	0.25	25	55	25	2.54	65.63	N
S052	BC	0.60	25	64	24	3.16	64.02	Y
S053	OC	0.56	29	58	7	1.94	63.93	Y
S054	OC	0.79	34	21	35	1.69	63.81	Y
S055	OC	0.59	24	22	66	3.16	63.02	Y
S056	OC	0.65	26	11	86	3.97	61.47	N
S057	BC	0.79	26	44	20	1.90	60.55	Y
S058	OC	0.38	27	18	65	3.20	60.40	Y
S059	OC	0.63	27	17	44	1.81	59.95	N
S060	OC	0.45	29	17	38	1.71	57.50	Y
S061	OC	0.29	25	19	61	3.06	57.57	Y
S062	BC	0.69	30	33	18	1.55	56.98	Y
S063	OC	0.64	25	20	62	3.25	56.63	Y
S064	OC	0.67	31	16	54	2.90	56.05	Y
S065	BC	0.20	29	48	13	2.20	55.90	N
S066	OC	0.55	27	23	43	2.39	55.96	N
S067	OC	0.78	24	19	47	2.28	54.66	N
S068	OC	0.46	22	24	38	1.90	54.55	Y
S069	OC	0.51	28	18	57	3.16	54.02	N
S070	OC	0.74	23	18	40	1.75	53.88	Y
S071	OC	0.50	25	15	44	1.99	53.16	N
S072	OC	0.33	30	17	43	2.43	52.34	Y
S073	OC	0.47	15	20	69	3.34	52.23	Y
S074	OC	0.50	25	19	35	1.84	50.48	N
S075	OC	0.80	24	14	44	2.05	50.23	N
S076	OC	0.36	19	16	65	3.29	49.01	N
S077	BC	0.64	26	38	15	1.95	48.78	Y
S078	OC	0.53	24	14	38	1.77	48.57	Y
S079	OC	0.82	24	18	34	1.79	48.26	N
S080	BC	0.56	23	35	18	1.82	47.79	Y
S081	OC	0.56	25	17	32	1.72	47.34	Y
S082	OC	0.88	23	19	32	1.74	47.03	Y
S083	OC	0.77	11	20	74	3.74	47.03	N
S084	OC	0.37	25	19	32	1.88	46.86	N
S085	OC	0.74	17	19	61	3.28	46.16	Y

S086	OC	0.73	25	16	38	2.14	45.83	Y
S087	OC	0.74	27	14	37	2.08	45.76	Y
S088	OC	0.76	24	14	34	1.72	45.34	Y
S089	BC	0.33	26	31	13	1.61	45.05	N
S090	OC	0.47	24	17	49	2.91	44.90	Y
S091	OC	0.62	23	15	36	1.88	44.86	N
S092	OC	0.32	24	19	45	2.79	44.76	Y
S093	OC	0.62	23	12	37	1.82	43.79	N
S094	BC	0.77	23	34	17	1.96	43.62	Y
S095	OC	0.46	19	17	36	1.90	42.55	N
S096	OC	0.37	21	18	51	3.12	41.64	Y
S097	OC	0.83	23	12	34	1.77	41.57	N
S098	OC	0.82	22	17	32	1.92	41.24	N
S099	OC	0.66	19	17	49	2.86	40.67	Y
S100	OC	0.24	24	16	36	2.30	40.35	N
S101	OC	0.61	17	18	54	3.14	40.33	Y
S102	OC	0.65	21	12	37	1.94	39.93	N
S103	OC	0.86	22	16	29	1.77	39.57	Y
S104	OC	0.66	22	20	46	3.15	39.18	Y
S105	BC	0.44	23	26	17	1.76	38.72	N
S106	OC	0.32	11	10	56	2.49	38.41	Y
S107	OC	0.29	22	13	30	1.74	38.03	N
S108	OC	0.79	23	20	37	2.72	37.84	N
S109	OC	0.64	21	16	30	1.92	37.24	N
S110	OC	0.70	19	16	29	1.75	36.88	N
S111	OC	0.26	22	13	31	1.90	36.55	N
S112	OC	0.56	21	15	27	1.72	36.34	Y
S113	OC	0.54	13	19	42	2.45	36.03	N
S114	OC	0.43	20	15	29	1.84	35.48	N
S115	OC	0.71	21	12	32	1.92	35.24	Y
S116	BC	0.48	18	55	18	3.62	34.89	Y
S117	OC	0.31	22	14	30	2.05	34.23	Y
S118	OC	0.79	16	19	29	1.93	34.09	Y
S119	OC	0.65	23	13	25	1.81	32.95	Y
S120	BC	0.43	17	38	21	2.78	32.91	N
S121	OC	0.63	18	11	37	2.16	32.52	N
S122	OC	0.44	17	6	51	2.72	31.84	Y
S123	OC	0.53	16	14	38	2.34	31.73	Y
S124	OC	0.68	15	14	32	1.89	31.71	N
S125	OC	0.63	14	13	47	2.74	31.53	N
S126	OC	0.21	17	9	42	2.37	31.27	N
S127	OC	0.20	20	11	36	2.37	30.27	Y
S128	OC	0.78	22	12	22	1.67	30.12	N
S129	OC	0.52	18	13	26	1.80	29.10	N

S130	OC	0.67	19	12	27	1.87	29.02	N
S131	OC	0.46	16	17	26	1.94	28.93	N
S132	OC	0.44	17	23	36	3.06	28.57	N
S133	OC	0.67	18	17	42	3.16	28.02	N
S134	BC	0.40	14	28	17	2.00	28.00	N
S135	OC	0.70	9	17	53	3.35	27.08	N
S136	OC	0.36	16	13	25	1.75	26.88	N
S137	OC	0.27	19	14	22	1.87	26.02	N
S138	OC	0.40	16	11	43	2.84	25.98	N
S139	OC	0.56	16	15	24	1.89	25.71	N
S140	OC	0.85	19	9	19	1.41	25.15	N
S141	OC	0.60	19	9	25	1.87	24.02	N
S142	OC	0.46	14	13	37	2.59	23.86	N
S143	OC	0.23	18	10	38	2.02	34.68	Y
S144	OC	0.30	11	51	5	2.79	23.76	N
S145	OC	0.78	14	14	26	1.96	23.62	N
S146	OC	0.40	15	7	26	1.59	23.36	N
S147	OC	0.84	16	13	22	1.80	23.10	N
S148	BC	0.27	14	24	16	1.99	23.16	Y
S149	BC	0.41	8	61	15	3.99	22.16	N
S150	OC	0.36	16	13	22	1.90	21.55	N
S151	OC	0.88	17	9	37	2.73	20.69	N
S152	OC	0.42	16	11	26	2.11	20.30	N
S153	OC	0.50	16	16	43	3.55	19.98	N
S154	BC	0.35	16	33	15	2.92	18.74	N
S155	BC	0.48	14	26	11	2.15	17.68	N
S156	OC	0.41	15	16	37	3.25	17.63	N
S157	OC	0.67	16	12	19	1.96	16.62	N
S158	BC	0.43	13	22	12	1.97	16.47	N
S159	OC	0.47	12	13	34	2.83	15.14	N
S160	OC	0.86	15	7	19	1.68	14.96	N
S161	BC	0.38	14	24	8	2.10	13.45	N
S162	OC	0.41	13	14	41	3.52	13.44	N
S163	OC	0.76	16	12	14	1.86	13.17	N
S164	BC	0.36	16	18	6	1.74	13.03	N
S165	OC	0.88	14	7	19	1.75	12.88	N
S166	OC	0.60	8	15	42	3.38	12.61	N
S167	OC	0.78	5	14	40	3.15	10.18	N
S168	OC	0.25	15	10	19	2.19	10.06	N
S169	OC	0.54	15	5	23	2.13	9.99	N
S170	OC	0.41	11	16	33	3.23	9.94	N
S171	OC	0.21	11	9	28	2.47	9.72	N
S172	OC	0.77	5	6	31	2.21	7.75	N
S173	BC	0.46	11	16	9	1.85	7.33	N

S174	OC	0.82	14	11	31	3.19	6.56	N
S175	BC	0.20	13	12	19	2.43	6.34	N
S176	BC	0.47	13	15	6	1.79	6.26	N
S177	OC	0.36	9	12	18	2.15	5.68	N
S178	BC	0.38	11	15	9	1.92	5.24	N
S179	OC	0.71	6	9	41	3.41	3.15	N
S180	OC	0.53	8	15	33	3.42	2.99	N
S181	OC	0.37	8	11	36	3.40	2.30	N
S182	BC	0.41	9	17	6	2.05	0.23	N
S183	OC	0.64	7	12	28	3.03	0.04	N
S184	OC	0.80	12	5	14	2.01	-0.15	N
S185	OC	0.63	12	6	15	2.17	-0.63	N
S186	OC	0.28	11	8	17	2.59	-4.15	N
S187	BC	0.32	7	16	4	2.04	-4.62	N
S188	OC	0.64	4	7	40	3.69	-6.20	N
S189	BC	0.60	6	11	7	1.98	-6.69	N
S190	BC	0.35	4	17	7	2.50	-10.75	N
S191	OC	0.35	6	4	8	1.93	-11.92	N
S192	OC	0.26	6	5	7	2.10	-14.55	Y
S193	OC	0.63	2	11	32	3.86	-14.83	N
S194	OC	0.77	3	3	4	2.10	-22.55	N
S195	OC	0.83	0	3	24	4.07	-36.09	N

*Y means that BRCA1/2 is deficient, and N means that *BRCA1/2* is intact.

Supplementary Table 4 Tumor purity and ploidy values calculated by tumor cell lines and clinical samples with different tumor purity

ID	purity.pdt	purity.cal	ploidy.cal	HRD Score
HCC1143	0.80	0.83	3.47	61.27
	0.60	0.59	3.47	63.29
	0.50	0.50	3.44	61.71
	0.40	0.39	3.43	64.83
	0.30	0.29	3.35	66.02
	0.25	0.24	3.27	62.32
	0.20	0.20	3.31	54.64
	0.10	1.00	2.12	-22.80
HCC1428	0.80	0.80	3.61	30.97
	0.60	0.59	3.60	33.26
	0.50	0.48	3.59	32.33
	0.40	0.39	3.58	38.33
	0.30	0.29	3.58	32.39
	0.25	0.24	3.59	30.24
	0.20	0.19	3.44	24.15
	0.10	1.00	2.16	-21.55
HCC38	0.80	0.69	3.25	74.86
	0.60	0.57	3.35	76.10
	0.50	0.45	3.27	74.05
	0.40	0.35	3.29	70.57
	0.30	0.26	3.29	76.80
	0.25	0.22	3.36	72.26
	0.20	0.16	3.18	46.79
	0.10	1.00	2.00	-29.14
clinical_sample1	0.47	0.55	2.60	50.16
	0.31	0.36	2.60	47.94
	0.16	0.19	2.30	19.36
clinical_sample2	0.44	0.46	2.90	2.82
	0.29	0.32	2.80	6.84
	0.15	0.20	2.40	-8.38
clinical_sample3	0.27	0.32	2.00	38.68
	0.18	0.20	2.00	32.09
	0.09	0.09	2.10	-18.15
clinical_sample4	0.25	0.29	3.80	12.19
	0.17	0.20	3.90	8.43
	0.08	0.10	4.00	-7.16
clinical_sample5	0.33	0.34	1.90	70.01
	0.22	0.23	1.80	69.59
	0.11	0.14	2.00	-1.02

Supplementary Table 5 Comparison of the chromosome segmentation and copy number of GSA, PureCN, and ASCAT.

GSA					PureCN				ASCAT				
chromosome	start	end	nA	nB	chromosome	start	end	Copy number	chromosome	start	end	nMajor	nMinor
1	10443	3683916	5	5	1	10544	108910866	3	1	10444	255957	2	0
1	3683916	16797479	2	2	1	110246770	121350186	3	1	534242	1509825	14	8
1	16797480	22451377	3	3	1	121390914	142728945	6	1	1534614	3683916	7	6
1	22451378	74054262	2	2	1	142811022	228198520	3	1	3686315	5748792	3	3
1	74054263	78149691	2	3	1	228617059	231790931	3	1	5765147	6695123	6	6
1	78149692	120383827	2	2	1	231790932	249239442	2	1	6700095	16054700	4	3
1	120383828	150059069	2	3	2	10287	20845260	3	1	16063698	17331676	9	6
1	150059069	164109310	2	2	2	20870815	89560394	3	1	17342924	33851400	4	3
1	164109311	177669266	1	2	2	91757142	146858894	3	1	33858796	40768552	3	3
1	177669267	201016435	2	2	2	146885790	240957786	3	1	40769415	45297753	4	3
1	201016436	206694171	2	3	3	105785	97983265	3	1	45307506	51705962	3	2
1	206694171	249239610	2	2	3	97983266	129279827	3	1	51730054	55418976	3	3
2	12990	75590677	1	2	3	129331896	183907028	3	1	55429134	55538385	7	6
2	75590677	173916192	2	2	3	184312097	197860922	2	1	55541174	74379335	3	2
2	173916193	177912470	2	3	3	184312097	197860922	2	1	74388971	77828321	4	2
2	177912471	241066575	2	2	4	10798	9071586	3	1	77840200	85036239	2	2
2	241066575	243184927	4	4	4	9093573	15004879	4	1	85040069	85746754	5	3
3	105785	22966271	1	2	4	15009222	69382795	3	1	85768379	109650386	3	2
3	22966271	25149971	1	3	4	70223753	190939656	3	1	109656420	110168888	6	4
3	25149971	31998548	1	2	5	2909107	42674475	3	1	110169957	110254678	8	5
3	31998549	35044116	1	3	5	42674476	100669673	3	1	110268828	113123734	3	3
3	35044117	64619211	1	2	5	100669674	180719384	3	1	113140583	113290718	6	4

3	64619212	67527113	1	3
3	67527114	89232164	1	2
3	89232165	164773057	2	2
3	164773057	197861082	2	3
4	11870	3789961	2	2
4	3789961	190907201	1	1
5	11788	2413820	2	6
5	2413820	45414358	1	3
5	50329524	59284115	1	1
5	59284116	64758380	1	2
5	64758381	100730500	1	1
5	100730501	137802107	1	2
5	137802108	140959743	2	2
5	140959744	180719407	1	2
6	184125	122114448	1	1
6	122114448	125597537	1	2
6	125597537	171049459	0	1
7	34918	2758932	2	4
7	2758932	88774612	1	2
7	88774613	91497419	1	3
7	91497420	147891107	1	2
7	147891108	159127624	1	3
8	11892	31255894	1	2
8	31255895	36469972	1	1
8	36469973	39694832	1	2
8	39694833	139768085	2	2

6	183913	29855898	3
6	34078428	125368280	3
6	125368281	166718428	3
6	166736361	170978749	3
7	67812	54497365	3
7	57698751	61551813	3
7	61751405	76144500	3
7	76198922	84604610	3
7	88213051	118686043	3
7	118884560	141786093	3
7	143036338	147182357	3
7	147182358	159128528	3
8	10470	3172400	4
8	3190640	7222674	4
8	7868986	11878174	4
8	12575672	37190826	4
8	39390215	115234266	3
8	115240752	129073961	2
8	129077999	140789846	2
8	142021694	146301295	2
9	10308	20603357	3
9	20626651	28761500	4
9	28761501	95708348	3
9	96091676	127201626	3
9	129377847	141069049	3
10	2324863	51874242	3

1	113310636	120256713	3	2
1	120267544	121449630	5	1
1	142540154	143505099	3	1
1	143509346	156206115	4	3
1	156213912	156909303	5	5
1	156914070	164182915	3	2
1	164205963	177698891	2	1
1	177707062	185487243	3	2
1	185500360	197407942	2	2
1	197414077	200838925	3	3
1	200842104	200956288	7	6
1	200958111	201116018	9	8
1	201122947	201358297	6	5
1	201359717	204397427	4	3
1	204403659	205499698	7	4
1	205505474	225927179	2	2
1	225955919	227913886	3	2
1	227920061	228612838	6	5
1	228617063	228699477	3	2
1	228714745	241728046	2	2
1	241755341	242109146	5	2
1	242122950	249239246	2	2
2	10437	3605468	3	2
2	3610367	29183332	3	1
2	29205451	29338016	6	3
2	29347233	43927509	2	1

8	139768085	142199400	3	4	10	51911792	69482430	3	2	43931321	44099433	8	2
8	142199400	146300855	5	5	10	69528410	73782236	3	2	44101538	44502788	9	2
9	10345	30951193	1	1	10	73782237	79370711	3	2	44504877	75590677	2	1
9	30951194	125570106	1	2	10	79403929	103501765	3	2	75612112	95847037	3	2
9	125570107	129864419	2	2	10	103501766	133218174	3	2	95862265	98413053	4	3
9	129864420	133967240	2	4	11	193151	3315255	3	2	98419726	113817566	3	2
9	133967240	136119809	1	2	11	3362845	8657322	4	2	113819614	114035194	4	4
9	136119809	141034077	3	5	11	8707150	15267597	3	2	114044377	120436681	3	2
10	135708	2989837	1	1	11	15267598	64012335	3	2	120438515	121854242	4	3
10	2989837	7063049	1	5	11	70605729	77069740	3	2	121873228	126322442	2	2
10	7063050	38181470	1	2	11	77111279	124249831	3	2	126335702	130860632	3	2
10	42546687	47006215	1	3	11	124249832	134946332	3	2	130870631	132302165	4	4
10	47006216	72990780	1	2	12	193817	9619771	3	2	132312325	173884290	2	2
10	72990781	77392753	2	5	12	9745645	14705349	3	2	173913258	178417225	3	3
10	77392754	103535470	1	3	12	14748352	24215922	3	2	178430747	216863221	2	2
10	103535471	132854084	2	3	12	26581301	54410000	3	2	216878215	218674353	4	3
10	132854084	135523863	5	7	12	54756526	133839682	3	2	218675144	220537405	4	4
11	193096	3254265	0	5	13	19365400	115108995	3	2	220543947	233272520	3	2
11	3254265	15287427	0	2	14	20369396	41602932	3	2	233273244	233410503	8	7
11	15287428	63668557	1	2	14	41676644	73181555	3	2	233415520	238166147	3	3
11	63668557	65906059	4	4	14	74040334	82491999	3	2	238172570	238503559	6	4
11	65906060	71329189	2	4	14	82503803	106412532	3	2	238509769	243184927	5	4
11	71329189	117054709	1	2	15	24752517	30432873	3	3	105785	9798006	2	1
11	117054709	120387112	2	3	15	30889846	93588316	3	3	9798767	9991369	4	3
11	120387112	134946381	1	2	15	93616927	102431165	3	3	10015437	12854616	2	1
12	193817	44325870	1	2	16	2998646	32096230	3	3	12856856	13726888	4	2

12	44325871	52856128	1	3
12	52856129	69518950	1	2
12	69518950	72779820	2	3
12	72779820	130897316	1	2
12	130897316	133840496	2	3
13	19149491	35918811	1	2
13	35918812	38039200	1	3
13	38039200	97966870	1	2
13	97966871	100749907	1	3
13	100749908	115104231	1	2
14	19066305	104518453	1	2
14	104518453	107289541	2	4
15	20119124	95416418	1	1
15	95416419	102520957	1	2
16	103517	5196763	2	2
16	5196763	28336882	1	1
16	28336882	33965813	2	2
16	33965813	69525862	1	1
16	69525862	72832651	0	2
16	72832651	87518783	1	1
16	87518783	90230101	2	2
17	2037	8851027	0	3
17	8851027	33194247	0	2
17	33194248	45664593	0	1
17	45664593	48761099	1	3
17	48761099	69119481	1	2
16	33839127	55778841	3	
16	55832532	90171440	3	
17	301	15039064	3	
17	18221011	21982191	3	
17	21982192	26633385	3	
17	27816679	36211682	3	
17	38140545	45490251	3	
17	45490252	81050912	3	
18	10718	77674628	4	
19	844021	14924875	3	
19	24487371	38793310	2	
19	41104875	57804362	3	
20	76963	9806342	3	
20	9836329	26072253	3	
20	26278201	29652157	4	
20	29809688	33585427	3	
20	33591112	38747958	3	
20	39998485	52169749	2	
20	55892520	62916447	2	
21	9527025	14572806	4	
21	14578435	40683477	3	
21	40712373	48118698	3	
22	24681539	30638282	3	
22	30802434	51243277	3	
3	13734613	23503506	2	1
3	23511338	24830136	3	1
3	24838218	31981493	2	1
3	32001641	35035432	4	1
3	35045130	38034821	2	1
3	38035508	38793940	3	2
3	38802251	48384721	2	1
3	48393556	48678011	5	3
3	48678971	48723302	7	4
3	48727112	49928691	3	1
3	49936102	50645793	7	3
3	50647888	51697493	2	1
3	51737965	52512318	4	2
3	52521921	52584424	7	4
3	52584715	53277645	3	2
3	53293157	65306638	2	1
3	65311745	67425983	4	1
3	67431071	88916705	2	1
3	88926656	123005476	3	2
3	123010363	123135244	5	5
3	123152897	123419733	5	4
3	123440967	123457711	8	7
3	123479778	125962089	3	2
3	125975564	129349136	4	4
3	129364254	147066494	3	2
3	147076030	147599269	4	4

17	69119481	78765219	2	4
17	78765219	81194987	2	5
18	10813	78017073	0	2
19	282747	4818373	0	2
19	4818373	14231330	2	6
19	14231330	17296679	1	2
19	17296679	20184274	2	4
19	20184274	22482889	1	4
19	22482890	38604588	1	1
19	38604588	41284394	2	2
19	41284394	48785642	1	4
19	48785642	56767042	2	4
19	56767042	59118886	1	2
20	76962	25041159	2	2
20	30273552	49774398	2	2
20	49774399	53291424	2	4
20	53291425	56433748	2	3
20	56433749	60180124	2	2
20	60180124	62958710	4	4
21	9527088	32935103	2	2
21	32935103	35506430	2	3
21	35506430	43852421	2	2
21	43852422	48104004	4	4
22	16870802	24655659	0	3
22	24655659	28604583	0	2
22	28604583	31438928	0	4

3	147623909	164764719	2	2
3	164773057	175513789	4	3
3	175520406	183907032	3	3
3	183914759	184311940	7	7
3	184320458	184485565	5	4
3	184492965	193798407	3	3
3	193807621	197861082	5	4
4	10798	515489	1	1
4	515844	2074700	3	2
4	2087202	2307960	1	1
4	2330889	3789961	2	1
4	3798866	6525332	1	1
4	6532811	9022171	2	1
4	9032235	190939879	1	0
5	12114	14104	9	0
5	14757	49851	3	1
5	50708	1889840	8	3
5	1902586	42779736	3	1
5	42782492	43855368	4	3
5	43927178	45387846	3	1
5	45406321	49505838	2	1
5	49522346	59277134	1	0
5	59286205	64693317	2	1
5	64725524	79238533	1	0
5	79252182	79617357	2	1
5	79621016	94259610	1	0

22	31438928	51183255	0	2

5	94278129	94528491	2	1
5	94550971	100753611	1	0
5	100765147	138487483	2	1
5	138494690	138705812	2	2
5	138713959	141335241	4	3
5	141336264	148152364	2	1
5	148164206	148581063	6	2
5	148610276	149431259	3	1
5	149433596	150158164	4	2
5	150164243	167653394	2	1
5	167674363	168634740	6	2
5	168671698	175956516	2	1
5	175956516	176148768	6	3
5	176165086	180719407	4	2
6	184125	25918688	1	0
6	25944103	33272712	1	1
6	33280505	34110254	2	1
6	34124370	44691371	1	1
6	44698708	90565234	1	0
6	90572196	91204213	2	1
6	91225132	98280013	1	0
6	98300239	98749236	2	1
6	98771028	122605510	1	0
6	122617158	124201918	2	1
6	124204860	125572968	4	1
6	125597537	166554170	0	0

6	166572045	171050287	1	0
7	16165	2458380	5	2
7	2472707	3117838	7	3
7	3127215	5239360	3	1
7	5254227	5686704	6	3
7	5696125	6870635	3	1
7	6878096	43846603	2	1
7	43851539	45257233	4	2
7	45273264	54133053	2	1
7	54142421	54689570	2	2
7	54711985	72286028	2	1
7	72300323	72891754	3	2
7	72898228	72934078	3	0
7	72944370	76162076	4	2
7	76173536	89563399	2	1
7	89574638	91443183	4	1
7	91464218	98990416	2	1
7	99017733	102312228	3	2
7	102332448	147821399	2	1
7	147839308	149321637	4	2
7	149334780	149523584	11	4
7	149528146	149576819	10	4
7	149589663	159128605	5	2
8	10470	2226147	3	1
8	2239128	10438796	2	1
8	10448836	10649192	5	3

8	10656437	16059915	2	1
8	16066975	17581155	3	1
8	17581700	19221650	2	1
8	19237079	20110641	3	2
8	20120297	21862551	2	1
8	21862551	23290439	4	2
8	23291833	25460335	2	1
8	25481266	26988103	1	0
8	27001935	31352513	2	1
8	31388393	35916718	1	0
8	35922774	39685594	2	1
8	39694860	43763025	3	2
8	43776238	47346329	4	4
8	47357126	84030205	2	2
8	84038956	123445622	3	2
8	123465838	140611843	3	3
8	140630990	142129925	7	4
8	142138860	145921475	9	8
8	145933692	146301381	4	3
9	10345	57539	2	1
9	58033	31146953	1	0
9	31164389	95826971	2	1
9	95840344	96060759	6	3
9	96072783	116741937	2	1
9	116751005	117195760	5	3
9	117203632	125840164	2	1

9	125848900	129551392	3	3
9	129558047	130210946	3	1
9	130213298	134530107	4	2
9	134541216	135841425	2	1
9	135861993	141069049	5	3
10	135708	1782394	1	1
10	1795194	3009351	1	0
10	3022248	3188071	15	4
10	3189380	6881554	8	2
10	6900363	38958568	2	1
10	38968721	42517060	3	1
10	42607765	47738963	5	2
10	47898043	71119003	2	1
10	71129194	73439101	3	2
10	73454637	73579217	6	3
10	73581560	73782236	3	1
10	73790819	77359185	7	2
10	77370319	80898955	4	1
10	80912499	81198212	7	3
10	81212704	92791385	4	1
10	92806230	96163034	6	2
10	96176376	103501914	4	1
10	103510619	110903958	4	4
10	110913556	111946947	4	1
10	111956363	133482891	4	4
10	133503598	135279810	10	9

10	135280765	135523863	7	2
11	193096	279786	3	1
11	280216	1961869	5	0
11	1971977	2073490	9	0
11	2094852	2436733	8	0
11	2439621	2441646	9	0
11	2443938	3254265	4	0
11	3260222	15282156	2	0
11	15301762	60504476	2	1
11	60508999	64039162	3	2
11	64053595	65617110	7	6
11	65623524	70517927	4	2
11	70523008	75852448	3	1
11	75860179	76924874	4	2
11	76928149	77814045	3	1
11	77815188	102587062	2	1
11	102593248	102870940	2	2
11	102884896	116634173	2	1
11	116643610	118472349	3	2
11	118478407	118604837	5	3
11	118626511	118757219	3	2
11	118757219	120151774	6	3
11	120161671	125714088	2	1
11	125747537	125986274	3	3
11	125998594	126232422	4	4
11	126242546	126404639	6	5

11	126411459	134946318	2	1
12	193817	907310	3	2
12	928911	6422272	2	1
12	6422988	7287914	4	2
12	7290200	44604028	2	1
12	44613070	48920006	4	1
12	48925862	50480232	3	1
12	50480314	52204246	2	1
12	52215102	53822992	4	2
12	53825325	57559809	2	1
12	57567180	57591458	7	4
12	57591458	57637593	6	3
12	57640620	69654187	2	1
12	69667075	70679929	3	3
12	70690194	72203204	3	1
12	72226611	113369644	2	1
12	113376452	113996719	4	4
12	114005404	120443494	2	1
12	120455954	121202362	6	2
12	121202952	122692820	3	2
12	122701001	124777480	1	1
12	124787539	125621419	3	2
12	125626535	131455442	2	1
12	131462375	132505406	4	2
12	132508389	133464490	7	7
12	133488904	133841396	3	2

13	19110523	35933714	2	1
13	35955344	37915301	4	1
13	37925269	98218375	2	1
13	98234789	100804923	4	2
13	100807376	110759711	2	1
13	110770593	113411956	3	2
13	113421845	115108995	5	3
14	19066305	24437922	2	1
14	24454989	25008758	4	2
14	25026780	34014124	2	1
14	34020584	35019099	3	1
14	35032112	99659371	2	1
14	99669020	104554044	3	2
14	104559552	106404410	5	3
14	106412532	107289541	2	1
15	20060562	40329520	1	0
15	40330693	42083843	1	1
15	42104339	42226255	3	2
15	42233908	42431236	2	1
15	42433382	42448658	3	2
15	42453954	90170232	1	1
15	90172832	90352611	3	2
15	90369148	95706135	1	1
15	95712020	97706696	2	1
15	97712183	97875116	1	0
15	97888564	101162066	1	1

15	101172258	102520957	3	1
16	97583	3170188	3	2
16	3173575	4356193	1	1
16	4367492	5223036	2	2
16	5247799	11203558	1	0
16	11225515	11607076	2	2
16	11618256	27751757	1	1
16	27761631	29814234	2	1
16	29818850	30134251	5	3
16	30147265	35213861	2	1
16	46438402	56899547	1	1
16	56901288	58079157	2	1
16	58092093	66240435	1	0
16	66246688	70624483	1	1
16	70630077	71174927	2	1
16	71188623	83949853	1	1
16	83953124	84211465	2	1
16	84212571	84270741	3	3
16	84296118	87349529	1	1
16	87350773	90292760	2	2
17	301	9846633	3	0
17	9856734	16321029	1	0
17	16322676	18286762	3	0
17	18314913	21195039	2	0
17	21198799	21321955	5	1
17	21356435	33502973	2	0

17	33504465	36614524	0	0
17	36617210	37558900	2	0
17	37561613	45745468	1	0
17	45750462	48834023	4	2
17	48843437	69919968	2	1
17	69928346	71189182	6	2
17	71192873	72710796	3	2
17	72733082	78846909	4	2
17	78854377	81194987	6	3
18	10813	52294	4	0
18	52880	76951258	1	0
18	76961923	78017073	2	0
19	266023	4844149	2	0
19	4847874	5307420	24	5
19	5320087	7431287	8	2
19	7437583	8675108	28	5
19	8741614	10057878	6	1
19	10070954	10265312	3	2
19	10267266	14200930	8	2
19	14231330	14412411	3	1
19	14425502	17396549	3	2
19	17397479	17655505	11	4
19	17660300	18119418	4	2
19	18119744	18174729	5	3
19	18180447	18950636	9	3
19	18956698	20913512	3	2

19	20918419	22559123	6	2
19	22585517	33044716	1	0
19	33054934	36038390	1	1
19	36041030	36259467	2	2
19	36266776	36643483	4	2
19	36667932	37397596	1	1
19	37399404	38109777	1	0
19	38127250	41497280	2	1
19	41510102	43237759	6	2
19	43243218	45253101	5	2
19	45287674	45911320	11	7
19	45912000	48692874	5	2
19	48697961	48785642	3	1
19	48800299	49132634	12	5
19	49133761	49141532	7	3
19	49143025	49206603	8	4
19	49214748	51455556	6	3
19	51463845	54314585	7	2
19	54327568	55879872	4	2
19	55895579	56215171	26	7
19	56220504	56717353	6	2
19	56717353	58858224	2	1
19	58858676	59118886	6	2
20	76962	2728558	4	3
20	2730400	3236015	7	6
20	3245006	3629686	3	2

20	3640467	3684022	9	8
20	3686436	3911270	6	5
20	3917072	24923660	3	2
20	24929947	25865400	4	4
20	25873122	30870339	5	3
20	30887184	33551218	4	3
20	33565480	33592474	9	9
20	33593674	43933163	3	2
20	43942676	44691350	5	4
20	44697431	47606043	3	3
20	47626847	47919672	5	3
20	47935629	50177662	3	3
20	50182797	53268372	7	4
20	53276418	55955567	5	3
20	55969697	56412867	7	5
20	56420820	60180124	3	3
20	60183964	62961724	7	6
21	9527088	14703726	4	2
21	14716293	33057344	2	2
21	33069118	35531416	4	3
21	35543613	39899525	3	2
21	39912560	42938226	4	3
21	42942620	45096206	5	4
21	45107518	47639492	7	6
21	47641700	48118698	4	4
22	16870802	18859378	4	0

		22	18894046	20311131	7	0
		22	20353879	22049783	4	0
		22	22053880	28586674	2	0
		22	28604583	31535995	4	0
		22	31552702	37260123	2	0
		22	37268257	38485064	4	0
		22	38486738	50058338	2	0
		22	50085205	51243277	1	0

Supplementary Table 6 Comparison of the tumor purity and ploidy predicted by GSA, PureCN, and ASCAT.

ID	purity.pdt	GSA		PureCN		ASCAT	
		purity.cal	ploidy.cal	purity.cal	ploidy.cal	purity.cal	ploidy.cal
HCC1143	0.8	0.83	3.47	0.84	3.62	0.8	3.91
	0.6	0.59	3.47	0.62	3.66	0.65	3.85
	0.5	0.5	3.44	0.5	3.66	0.61	3.17
	0.4	0.39	3.43	0.45	3.59	0.53	3.03
	0.3	0.29	3.35	0.32	3.71	NA	NA
	0.25	0.24	3.27	0.32	2.2	NA	NA
	0.2	0.2	3.31	0.32	2.25	NA	NA
	0.1	NA	NA	0.2	2.31	NA	NA
HCC1428	0.8	0.8	3.61	0.88	3.63	0.98	3.79
	0.6	0.59	3.6	0.6	3.63	0.72	3.88
	0.5	0.48	3.59	0.49	3.63	0.64	3.66
	0.4	0.39	3.58	0.4	3.66	NA	NA
	0.3	0.29	3.58	0.37	1.9	NA	NA
	0.25	0.24	3.59	0.27	2.05	NA	NA
	0.2	0.19	3.44	0.21	2.1	NA	NA
	0.1	NA	NA	0.15	1.78	NA	NA
HCC38	0.8	0.69	3.25	0.71	3.39	0.83	2.52
	0.6	0.57	3.35	0.56	3.4	0.69	3.05
	0.5	0.45	3.27	0.44	2.48	0.61	2.74
	0.4	0.35	3.29	0.41	3.44	0.47	3.16
	0.3	0.26	3.29	0.32	2.59	NA	NA
	0.25	0.22	3.36	0.32	2.29	NA	NA
	0.2	0.16	3.18	0.15	1.9	NA	NA
	0.1	NA	NA	0.15	2.06	NA	NA
clinical_sample1	0.74	0.74	3.00	0.80	3.18	0.87	3.65
	0.44	0.46	2.90	0.50	3.18	0.58	3.14
	0.29	0.32	2.80	0.33	3.21	0.45	2.35
	0.15	0.20	2.40	0.17	2.31	NA	2.08
clinical_sample2	0.45	0.45	2.00	0.46	2.17	0.65	1.84
	0.27	0.32	2.00	0.32	2.15	0.48	1.67
	0.18	0.20	2.00	0.22	2.17	NA	2.21
	0.09	NA	NA	0.15	2.12	NA	4.19
clinical_sample3	0.42	0.42	3.90	0.52	4.26	NA	NA
	0.25	0.29	3.80	0.39	4.19	NA	NA
	0.17	0.20	3.90	0.34	3.88	NA	NA
	0.08	NA	NA	0.22	3.81	NA	NA
clinical_sample4	0.54	0.54	1.90	0.45	2.04	0.64	1.44
	0.33	0.34	1.90	0.31	2.02	0.97	3.19
	0.22	0.23	1.80	0.22	2.05	NA	2.37

	0.11	NA	NA	0.15	2.03	NA	4.26
